# Response of plant roots to mechanical environmental stimuli

**DOI:** 10.1093/hr/uhaf337

**Published:** 2025-12-08

**Authors:** Bateer Baiyin, Yue Xiang, Yang Shao, Jung Eek Son, Kotaro Tagawa, Mina Yamada, Satoshi Yamada, Qichang Yang

**Affiliations:** Research Center for Smart Horticulture Engineering, Chengdu National Agricultural Science and Technology Center, Institute of Urban Agriculture, Chinese Academy of Agricultural Sciences, Chengdu 610213, China; Research Center for Smart Horticulture Engineering, Chengdu National Agricultural Science and Technology Center, Institute of Urban Agriculture, Chinese Academy of Agricultural Sciences, Chengdu 610213, China; Yazhouwan National Laboratory, Sanya, Hainan 572025, China; Department of Agriculture, Forestry and Bioresources, Seoul National University, Seoul 08826, Republic of Korea; Faculty of Agriculture, Tottori University, Tottori 680-8553, Japan; Faculty of Agriculture, Tottori University, Tottori 680-8553, Japan; Faculty of Agriculture, Tottori University, Tottori 680-8553, Japan; Research Center for Smart Horticulture Engineering, Chengdu National Agricultural Science and Technology Center, Institute of Urban Agriculture, Chinese Academy of Agricultural Sciences, Chengdu 610213, China

## Abstract

The mechanisms underlying plant root response to mechanical environmental stimuli are crucial for plant growth, development, and environmental adaptation. In this review, we examine the mechanical environments encountered by plant roots, including the different types of mechanical stimuli they experience. We describe in detail the mechanisms that enable roots to perceive these stimuli and their modes of action. Unfavorable mechanical stimuli can cause roots to alter their growth patterns and rates. Morphologically, roots become thicker, enhancing their stress resistance. Mechanical stimuli influence the activity of hormones, including auxin and ethylene, which jointly regulate root growth. Auxin promotes cell elongation in roots, whereas ethylene can inhibit root growth under certain conditions. Plants modulate antioxidant enzyme activity and osmoregulatory substance accumulation to cope with environmental stress. We explored the molecular regulatory mechanisms underlying plant root adaptation to mechanical stimuli, including those involved in regulating genes and signal transduction pathways. Finally, we suggest future research directions, including an in-depth study of the multi-signal integration mechanism of roots and gene editing technology for improving plant adaptability. This review provides a basis for studying the interactions between plants and mechanical environments for plant adaptation and agricultural production.

## Necessity of understanding the response of plant roots to mechanical stimulation

Plant growth is highly susceptible to various environmental stimuli, which affect the growth state of plants. Therefore, plants have evolved adaptive mechanisms that allow them to actively adapt to environmental changes through metabolic activities to ensure survival and reproduction [68]. The environmental impacts faced by plants are divided into aerial and rootzone parts. The aerial environment includes light conditions, atmospheric temperature, wind speed, and air humidity; the rootzone environment includes soil temperature, soil moisture, nutrient solution concentration, substrate pH, and electrical conductivity. These environmental factors can act individually or together on the growth of crops, forests, and grasses. A deep understanding of the response mechanisms of plants to the environment and their reasonable adjustment can improve agricultural production and ecological protection. This can help increase the yield and quality of crops, ensure food security, promote the healthy growth of forests and grass vegetation, and maintain ecosystem balance and stability.

Interdisciplinary research methods have been widely applied in plant science and agricultural production [80]. Against this background, the impacts of physical environmental factors, such as flow fields [36], on plant growth have been investigated. Among these factors, the mechanical environment has a particularly strong impact on plant growth. The mechanical environment covers a variety of influencing factors, including natural wind disturbances, artificial excavation operations, and pruning behaviors [43]. These mechanical actions affect the metabolic processes and morphology of plants to varying degrees [84]. The aerial parts of plants are directly exposed to the air, making them convenient for observations and non-destructive testing. Therefore, research on the impact of the mechanical environment on aerial parts is relatively easy to conduct. However, directly observing the response of roots to mechanical stimulation is more difficult, posing challenges to in-depth research on the growth mechanism of the root zone under mechanical stimulation.

**Figure 1:**
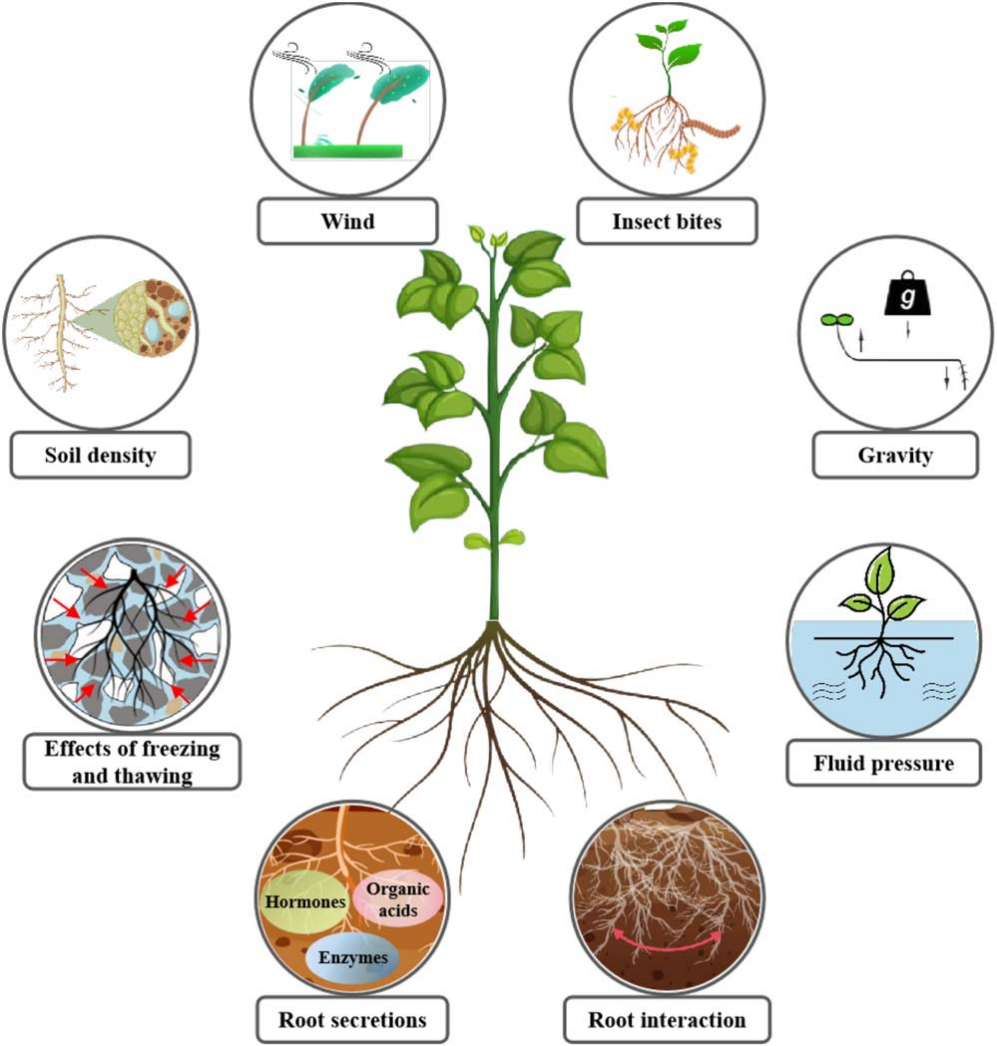
Key mechanical and environmental factors.

To date, through a large amount of research and data collation, it is clear that various mechanical factors in the environment affect roots (Table 1), and these factors can have direct or indirect effects on plant growth to varying degrees (Figure 1). Herein, we review the mechanisms underlying the response of plant roots to mechanical environments by comprehensively describing the changes that roots undergo when facing mechanical stimuli. Specifically, we explain the dynamic adjustment of root morphology, adaptive changes at physiological and biochemical levels, and associated signal transduction pathways. Our main objective is to provide a reference for future research on the interactions between plants and the mechanical environment. Therefore, we explored the significance of the response mechanisms for plant adaptation in agricultural production practices in the natural environment. This review provides theoretical support and practical guidance for the development of plant research and agricultural production.

**Table 1:** Mechanical environmental factors and their effects on roots.

**Mechanical Factor**	**Reference**	**Specific effects**	**Positive impacts**	**Negative impacts**
Gravity	[19]	Gravity continuously exerts a geotropic stimulus on plant roots.	Encourages roots to grow toward the ground, enabling the plants to take root firmly and contributing to overall uprightness and stability.	In special environments, such as the microgravity conditions in space, plants lose the normal gravitational stimulus. Thus, the growth direction of roots becomes disordered, affecting normal development and survival.
Soil compaction	([9]; [69])	Soil exerts varying degrees of mechanical resistance on roots.	Moderately compacted soil supports roots. It stimulates cell wall thickening, enhances stress resistance, encourages thicker roots, and improves anchoring ability.	High compaction increases the mechanical resistance exerted on the roots, which restricts their elongation and branching. Thus, growth slows, morphology gets distorted, and water and nutrient absorption are affected.
Obstacles (such as stones) in the cultivation substrate	([48]; [85])	Obstacles exert a reverse pressure on roots.	Stimulates thigmotropic growth response, prompting the roots to bypass the obstacles and continue growing. Thus, certain morphologies are formed, which enhance adaptability to complex environments.	Long-term contact with obstacles will restrict growth space, affect normal elongation and branching, lead to abnormal growth, and affect overall root function and plant growth and development.
Shrinkage and expansion during the freeze–thaw of soil	[93]	Soil volume change exerts alternating stimuli of extrusion and stretching on roots.	Can improve soil structure and increase porosity, thus creating better aeration and water-permeability conditions for root growth.	Frequent and intense freeze–thaw changes can directly damage root tissues and destroy cellular structure, impairing function. This affects the supply of water and nutrients to the plant, reduces cold and drought resistance, and may even lead to death.
Self-creep of soil	[75]	Slow soil movement exerts continuous and relatively weak lateral pressure and displacement stimuli on roots.	Slow creep can loosen soil, improve its aeration and water permeability, and optimize the micro-environment around the roots. Slight creeping stimuli may trigger adaptive growth mechanisms, prompting roots to secrete substances that help adapt to environmental changes and enhance stress resistance.	Prolonged soil creep can damage roots by causing them to deviate from their original growth direction, leading to root distortion and deformation, and damaging the conductive tissues, thus affecting the normal transportation of water and nutrients. To adapt to the constantly changing soil position, the roots need to consume extra energy for growth adjustment, which diverts the plant's energy from aboveground growth and reproduction.
Alternation of wetting and drying in soil	[77]	Change in pressure between soil particles caused by variations in soil moisture exerts stimuli on the roots.	Appropriate alternate wetting and drying can improve soil aeration, stimulate roots to grow deeper into the soil, increase the root-to-shoot ratio, and enhance the roots' ability to absorb water and nutrients.	If the we–dry alternation is too frequent or intense, roots are prone to damage during the repeated contraction and expansion, leading to breakage and affecting normal growth and development.
External force interference caused by the biting and wriggling of soil animals	([21]; [50])	Soil animal activities cause direct physical damage, while wriggling indirectly stimulates roots by altering soil micro-environment.	Nematodes use their stylets to pierce plant roots for feeding. When moving inside the roots, they cause squeezing and friction, which damages the root structure. Although mild stimulation may promote the colonization of beneficial microorganisms, nematodes inhibit the growth of root tips, cause abnormal root morphology.	Soil animals biting damages the epidermis and cortical tissues of roots, causing loss of protective barriers and making them vulnerable to pathogen infection. It affects conductive function, leading to obstructed water and nutrient transportation. Although insect wriggling can loosen the soil, it may disrupt the close contact between roots and soil, affecting nutrient absorption.
Biological trampling and animal digging	[1]	Trampling and digging exert direct mechanical stimuli such as extrusion and cutting on the roots.	In rare cases, light trampling may compact the soil to some extent, providing better support for the roots. However, this effect is limited.	It severely damages root structure and severs conductive tissues. Thus, plants are unable to absorb water and nutrients normally, which leads to stunted growth or even death. Even if the plants survive, they must consume a large amount of energy to repair the damaged roots, thus affecting their growth, development, and yield.

(Continued)

**Table 1:** Continued

**Mechanical Factor**	**Reference**	**Specific effects**	**Positive impacts**	**Negative impacts**
Entanglement among root systems	[13]	Roots generate mechanical stimuli through mutual extrusion and competition for space.	To a certain extent, the intertwining of roots can enhance the stability of the plant community and jointly resist external environmental stresses.	Root entanglement leads to intense spatial competition, which restricts the growth space of each plant's roots and affects the roots' ability to obtain water, nutrients, and oxygen.
Shaking and vibration from wind	([33]; [16])	Plants sway and vibrate, exerting indirect mechanical stimulation on roots.	Moderate wind-induced swaying stimulates root growth, promotes more developed roots, and enhances the lodging-resistance ability of plants.	Strong winds cause excessive shaking of plants, which may damage the connective tissues between the roots and the aboveground parts, thus harming the roots. In severe cases, plants may be blown down, resulting in root breakage, which affects survival and growth.
Flow of nutrient solution in hydroponics	[6]	Dynamic pressure stimulation on roots.	Appropriate dynamic pressure can stimulate growth and branching and promote nutrient absorption.	Excessive dynamic pressure from too high a flow rate will make the root structure compact, affect nutrient absorption, and is unfavorable for root growth.
Human harvesting activities and horticultural pruning	[57]	Direct stimuli, such as cutting and damaging roots.	Reasonable pruning can control root growth direction and density, regulate growth balance between the above- and underground parts, promote lateral root growth, make the root system more developed, and enhance the plant's ability to absorb nutrients and water.	Under improper practices, the root system will be severely damaged, which will affect the plant's regeneration ability and the growth in the next season. Excessive root pruning will harm the plants, leading to weak growth and affecting normal development and yield.

## Morphological and structural responses of plant roots to mechanical environmental stimuli

### Root morphology

When taproots grow in an environment with high mechanical resistance, such as compacted soil, their elongation is inhibited. As roots must overcome greater resistance when penetrating the soil, the elongation and division activities of cells are restricted. In contrast, in loose growing media, taproots grow downward more smoothly and usually grow longer [46].

The mechanical environment can affect the initiation and distribution of lateral roots. In roots, extrusion can induce the initiation and development of lateral root primordia, thereby increasing the number of lateral roots. Moreover, the growth direction of lateral roots is influenced by mechanical factors [88]. The roots grow in the direction with less resistance to better explore the soil space and acquire water and nutrients.

In stable and loose soil, roots may exhibit a relatively regular and dispersed branching pattern that allows them to fully utilize the soil space [78]. However, in an environment subjected to frequent disturbance or uneven resistance, root branching may become more complex and denser, forming a net-like structure. This can enhance the contact area between the roots and soil, thereby improving root anchoring ability.

Unlike soil cultivation, in hydroponics, the roots are not affected by the compactness of the cultivation substrate. However, the flow of the nutrient solution can be regarded as a type of mechanical stimulation [3]. The flowing liquid exerts mechanical stimulation on the roots, resulting in a greater total length and surface area than those of the roots growing under conditions with no flow or high flow [2]. This implies that mechanical stimulation can also be positive (eustress) [5]. Applying reasonable mechanical stimulation to plants is beneficial for the positive regulation of root growth and morphological development.

### Cell Wall structure and composition

To adapt to mechanical pressure, the walls of root cells thicken, enhancing cell strength and rigidity and enabling the root system to withstand greater external forces [25]. Lignin enhances the strength of the cell wall, and the degree of lignification responds to the mechanical environment. When the root system encounters mechanical resistance, large amounts of lignin accumulate in the cell wall, resulting in increased root lignification [97]. For example, as a tree ages and is influenced by the soil environment, the degree of root lignification gradually increases to support the overall tree structure and resist external mechanical interference. Lignified roots are tougher and better able to resist compression and friction [11].

The cortex structure is also influenced by the mechanical environment. In a compact substrate, the cortical cells of the root system may become smaller and more closely packed with reduced intercellular spaces, enhancing the ability of the roots to resist pressure and helping reduce friction when the roots penetrate the soil. In a loose substrate, the cortical cells are larger, and the intercellular spaces are wider, which helps the root system to absorb and transport water and nutrients [60].

In some environments that are frequently disturbed by external forces, such as hydroponic environments with flowing nutrient solutions, plant roots become more flexible [6, 7]. Under such dynamic conditions, roots do not need to anchor the plant as they do in soil. Instead, they need to sway flexibly to adapt to water flows with constantly changing directions. To better cope with this special mechanical environment, plant roots actively adjust their own physiological mechanisms to promote the synthesis and accumulation of cellulose and hemicellulose [2]. During this process, the ratio and arrangement of cellulose and hemicellulose may be adjusted to endow the cell walls with more suitable flexibility and elasticity [23]. Thus, the entire root system can better move with the water flow, avoiding breakage due to excessive rigidity when subjected to external forces.

### Three-dimensional configuration remodeling of roots

The mechanical environment can influence the proportion of photosynthetic products allocated to roots and shoots. When root growth encounters resistance, plants allocate more photosynthetic products to the roots to promote root growth and development, enhancing adaptability to adverse mechanical environments, thereby increasing the root–shoot ratio [89]. Within the same root system, biomass allocation to different parts also varies with the mechanical environment. When subjected to uneven mechanical stimuli, the root system preferentially accumulates biomass in parts that are under greater pressure or resistance. For instance, when the root system is blocked by an obstacle, the roots can increase biomass by increasing cell division and growth to bypass the obstacle and continue to grow [87].

A shallow hardpan restricts the vertical growth of roots, leading to increased horizontal expansion of root systems to find more suitable spaces for growth and obtain water and nutrients [98]. For plants growing on slopes, gravity and the mechanical properties of the soil prompt roots to adjust their spatial distribution. To fix the plant and resist downslope forces, roots are more developed on the lower side of the slope, with increased biomass and branching on this side to enhance plant support and anchorage [15].

In soils with an evident hierarchical structure, roots show a stratified growth pattern. Different soil layers have different physical properties, water content, and nutrient distribution. Roots adjust their growth and distribution to suit the conditions of each soil layer according to these differences [47]. This stratified structure enables roots to more effectively utilize resources in different soil layers and adapt to complex mechanical and chemical stimuli [95].

### Root Gravitropic growth

Gravity is a mechanical factor that can affect plant growth. Under normal gravity conditions, plant roots exhibit gravitropism; that is, roots grow in the direction of gravity. This is because root cap cells contain statoliths that can sense the direction of gravity [53]. The distribution of auxin in root cells changes through a series of signal transduction pathways, resulting in root growth toward the direction of gravity. This characteristic helps roots to penetrate deep into the soil, fix plants, and obtain water and nutrients from the deeper soil.

The gravitropic growth pattern of plant roots is disrupted in microgravity environments, such as outer space. Under such conditions, owing to the lack of an obvious gravitational direction, statoliths cannot sense specific gravitational information [73]. The distribution of auxin in root cells no longer follows the pattern regulated by gravity on the ground, causing the roots to grow in seemingly random directions, resulting in various irregular growth forms [82]. The roots of plants cultivated in a microgravity environment grow in a dispersed and disordered state as they cannot extend in the direction of gravity as they do on the ground. This unordered growth poses a challenge for plant cultivation in space [29]. Scientists should explore new methods to guide root growth to ensure normal plant growth and development in microgravity environments.

### Root Thigmotriposim

When roots come into contact with surrounding objects, they elicit a thigmotropic response. When encountering an obstacle, the roots change their growth direction and continue to grow around it. This process involves the perception and conduction of mechanical signals and a response mechanism that regulates cell growth [37]. For example, when roots encounter hard objects, such as stones, during growth, they adjust their growth direction and pass through the edges or gaps of the obstacle to avoid excessive compression and damage. In terms of mechanical signal perception, when roots encounter obstacles, their cell walls deform, triggering cellular responses. Conformational changes in mechanosensitive receptor proteins on the cell wall, such as extensin-like proteins, activate downstream signal transduction pathways. This leads to changes in intracellular electrical signals and rearranges the cytoskeleton (microfilaments and microtubules). Microfilaments transmit mechanical forces, while microtubules maintain cell shape and polarity. For example, the microfilaments in the front-end cells of roots will rapidly polymerize and depolymerize to adapt to pressure.

Regarding chemical signal perception, mechanical stimulus alters ion concentrations, particularly that of calcium ions [86]. When roots experience mechanical stress, calcium ion channels on the cell membrane open, thereby increasing the intracellular calcium ion concentration. This activates calcium-binding proteins to regulate downstream pathways and gene expression. For instance, when the root tip cells of corn encounter obstacles, the calcium ion concentration increases within minutes [14]. Furthermore, the distribution of auxin, a plant hormone, changes when roots encounter obstacles. Auxin content on the side of the obstacle increases, inhibiting cell growth and promoting root bending [58]. Other hormones, such as ethylene and abscisic acid, interact with auxin to jointly regulate the process.

Roots can also perceive obstacles through electrical signals, which are generated upon contact as a result of the activities of ion channels and ion pumps on the cell membrane. When the front-end cells are under pressure, the proton pumps are activated, causing changes in the potential difference and generating action potentials, which rapidly transmit information through plasmodesmata, enabling the entire root system to respond quickly.

## Physiological and biochemical responses of plant roots to mechanical environmental stimuli

### Regulation of plant hormones

Plant hormones regulate the response of root systems to mechanical stimulation. They cooperate and influence each other to jointly regulate the growth, morphology, and physiological functions of roots, helping plants better adapt to mechanical stimulation. The following sections elaborate on the specific regulatory roles of several major plant hormones in this process.

#### Auxin

When subjected to mechanical stimuli, the synthesis, transport, and distribution of auxin in roots change [44]. When roots encounter an obstacle and are mechanically stimulated, auxin accumulates on the stimulated side, whereas its content on the other side is relatively low. As the effect of auxin on cell elongation is concentration-dependent, a relatively high concentration of auxin inhibits cell elongation, whereas a lower concentration promotes it [35].

Mechanical stimulation activates the auxin signaling pathway. The auxin receptor TIR1 recognizes and binds to auxin, prompting the ubiquitination and degradation of Aux/IAA proteins, thereby releasing ARF transcription factors [42]. The ARFs enter the nucleus to regulate the expression of downstream genes involved in processes such as cell elongation and cell-wall synthesis, thereby influencing root growth and morphological development to adapt to mechanical stimulation. In addition, auxin often collaborates with ethylene to regulate the root's response to mechanical stimulation [44]. Ethylene can affect the synthesis, transport, and signaling of auxin [96]. Under mechanical stimulation, ethylene synthesis increases, which promotes auxin transport to the stimulated area, enhancing the accumulation and effect of auxin in that region, and further regulating the growth direction and rate of roots.

#### Ethylene

Ethylene can reduce cell wall extensibility and limit cell elongation. When roots encounter mechanical resistance, such as compacted soil, mechanical stimulation induces the upregulation of genes related to ethylene synthesis, such as *ACS* and *ACO* [71]. As a result, more ethylene is synthesized, root elongation is inhibited, and the roots may thicken to enhance their resistance to pressure. An appropriate level of ethylene can promote the initiation and development of lateral root primordia and increase the number of lateral roots.

After ethylene binds to its receptor through the signal transduction pathway, it activates a series of downstream signaling molecules, such as CTR1 and EIN2. EIN2 transmits the signal to the nucleus, activates the EIN3/EIL1 transcription factors, and regulates the expression of related genes, enabling root cells to undergo physiological and morphological responses. For example, the composition and structure of the cell wall are altered to enhance root tolerance to mechanical pressure.

### Abscisic acid

Abscisic acid can increase the content of osmotic adjustment substances, such as proline and soluble sugars, in cells, elevate cell osmotic pressure, and enhance cell tolerance to water stress caused by mechanical pressure [12]. When roots are subjected to mechanical stimuli, such as compression, abscisic acid accumulation helps maintain cell turgor and normal physiological functions, ensuring root survival under adverse conditions.

Abscisic acid inhibits the elongation of root cells, thereby slowing the root growth rate. When responding to mechanical stimuli, this growth inhibition allows the roots to allocate more energy to strengthening their own structure and resistance [52]. Moreover, abscisic acid coordinates with hormones such as auxin and ethylene to maintain the balance of root growth and development.

#### Cytokinin

When roots are stimulated by slight mechanical friction, the cytokinin content increases, enhancing cell division in the root apical meristem. This provides more cells to maintain root growth and repair capabilities while adapting to the mechanical environment. Cytokinin interacts with auxin in regulating root growth. Auxin mainly promotes the elongation of the main root, while cytokinin promotes the occurrence of lateral roots and root branching. When roots encounter mechanical resistance, the relative cytokinin content increases, promoting lateral root formation, developing the root system, and enhancing its adaptability to the mechanical environment.

#### Jasmonic acid

Jasmonic acid has a complex regulatory effect on root growth that is concentration dependent. This effect depends on the growth stage and environmental conditions of the plant. Under mechanical stimulation, jasmonic acid (JA) induces the expression of genes encoding enzymes involved in cell wall synthesis and modification, such as cellulose synthase and pectin methylesterase [96]. By regulating the activities of these enzymes, JA can alter the composition and structure of the cell wall, thereby increasing its strength and toughness.

#### Gibberellin

Under normal circumstances, gibberellin promotes the elongation and growth of root cells. However, under mechanical stimulation, its synthesis and signal transduction may be inhibited. As mechanical stimulation often indicates a poor growth environment for roots, a decrease in the gibberellin level slows root growth, allowing the roots to allocate more energy to coping with mechanical stress, such as strengthening the cell wall. When the mechanical stimulation is removed or weakened, the gibberellin level may recover, and the root growth rate will increase accordingly. Gibberellin can enhance the promoting effect of auxin on cell elongation and cooperate with cytokinin to influence the division and elongation of root cells. When roots respond to mechanical stimulation, the balance and interaction among various hormones affect their final growth and adaptation strategies [8].

### Reactive oxygen species metabolism

Mechanical stimulation leads to the accumulation of reactive oxygen species (ROS) such as superoxide anions (O₂^-^) and hydrogen peroxide (H₂O₂) in the roots. Mechanical stimulation affects the normal metabolism of root cells, disturbing the electron-transport chains in organelles such as mitochondria and chloroplasts, thus generating excessive ROS [64]. To scavenge the excessive ROS, roots activate the antioxidant enzyme system, including superoxide dismutase, catalase, and peroxidase. Superoxide dismutase can disproportionate O₂^-^ into H₂O₂, while catalase and peroxidase are responsible for decomposing H₂O₂ into water and oxygen, thereby maintaining the dynamic balance of ROS within the cells [63]. Some non-enzymatic antioxidant substances, such as ascorbic acid and glutathione, accumulate in the roots. They can directly react with ROS, reducing the concentration of ROS and alleviating the damage of oxidative stress to root cells [6].

### Cell Wall remodeling

Plants modulate antioxidant enzyme activity and osmoregulatory substance accumulation to cope with stress. In addition to these physiological responses, the molecular regulatory mechanisms underlying plant root reactions to mechanical stimuli are equally crucial. The cell wall helps plant cells to resist mechanical stimulation. When the roots are subjected to mechanical stimulation, the composition and structure of the cell wall will change. For example, the contents of components such as cellulose, hemicellulose, and lignin increase, thereby enhancing cell wall strength and rigidity [24]. The activities of enzymes related to cell wall synthesis, such as cellulose synthase, lignin synthase, and pectin methylesterase, are also regulated by mechanical stimulation [25]. These enzymes participate in the synthesis and modification processes of the cell wall, and changes in their activities will lead to alterations in its components and structure [17].

**Figure 2:**
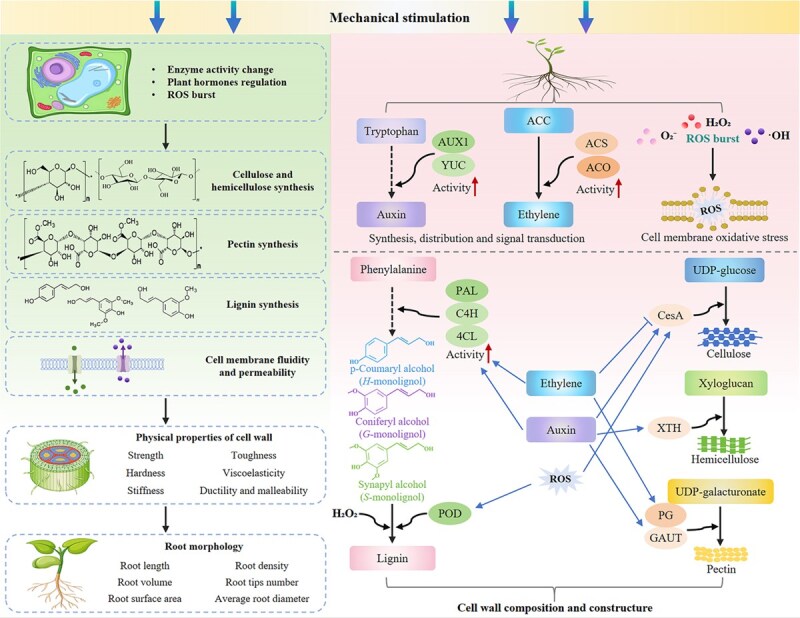
Response of plant roots to mechanical stimuli (physiological and biochemical perspectives). Mechanical stimuli can trigger changes in plant phenotypes at various levels, from microscopic to macroscopic, covering cells, tissues, organs, and even the whole plant. This figure illustrates the physiological and biochemical responses of plant roots to mechanical stimuli.

Mechanical stimuli can induce the enhanced activities of enzymes such as indole-3-pyruvate monooxygenase (YUC), auxin influx carrier auxin-resistant 1 (AUX1), 1-aminocyclopropanecarboxylic acid (ACC) synthase (ACS), and 1-aminocyclopropanecarboxylic acid oxidase (ACO), promoting the synthesis, distribution, and signal transduction of auxin and ethylene (Figure 2), which can promote ROS generation in cells and trigger oxidative stress responses. Mechanical stimuli may also induce changes in the activities of enzymes such as phenylalanine ammonia-lyase (PAL), cinnamate-4-hydroxylase (C4H), 4-coumarate-CoA ligase (4CL), peroxidase (POD), cellulose synthase (CesA), xyloglucan endotransglucosylase/hydrolase (XTH), polygalacturonase (PG), and galacturonosyltransferase (GAUT) to regulate the biosynthesis and accumulation of lignin, cellulose, hemicellulose, and pectin. This affects the content and structure of cell-wall components, thereby influencing the physical properties (e.g., strength, hardness, stiffness, toughness, and ductility) of the cell wall. Ultimately, root morphology changes to enable the roots to adapt to mechanical stimulation. Ethylene, auxin, and ROS also participate in the synthesis and modification of cell-wall components.

### Adjustment of metabolic pathways and accumulation of secondary metabolites

Mechanical stimuli may affect nitrogen absorption and assimilation in roots. In some cases, roots increase the absorption of ammonium nitrogen or nitrate nitrogen and enhance the activities of key enzymes in nitrogen metabolism, such as nitrate reductase and glutamine synthetase, to meet the needs of root growth and cope with stress [76]. Phenolic compounds possess antioxidant and antibacterial properties and enhance cell wall strength. The synthesis and accumulation of phenolic compounds increase when roots are subjected to mechanical stimuli. For example, flavonoids can scavenge ROS, protecting cells from oxidative damage, and the synthesis of lignin is closely related to phenolic metabolism, which can enhance the hardness and toughness of the cell wall and improve the ability of roots to resist pressure [51].

Arginine and polyamine contents in roots increase significantly under flowing conditions compared with those under non-flowing conditions [4]. Arginine is involved in regulating the physiological processes of roots and is a key metabolite in the nitric oxide (NO) signal transduction pathway. This indicates that the change in arginine content likely plays a key role in the plant's physiological response to the flowing environment by affecting NO signal transduction.

### Membrane system stability and functional adjustment

Mechanical stimulation can influence the lipid composition of the root cell membranes. When coping with mechanical stress, the proportions of components, such as phospholipids and glycolipids, in membrane lipids may also change [6], which helps regulate membrane permeability and the activity of membrane proteins. The functions of various cell membrane proteins, such as those associated with ion channels, transport, and receptors, are adjusted under mechanical stimulation. For example, mechanical stimulation can activate certain ion channels, altering the transmembrane transport of ions, thereby affecting the osmotic pressure, membrane potential, and signal transduction of cells [83]. Transport protein activity may also be regulated to ensure that the absorption and transport of nutrients and water in the roots can adapt to changes in the mechanical environment. Receptor proteins can sense mechanical signals and convert them into biochemical signals within the cell, thereby initiating physiological responses [45].

## Signal transduction pathways of plant roots in response to mechanical environmental stimuli

### Perception of mechanical signals

The elasticity and rigidity of cell walls help them to sense external mechanical stimuli. When roots are subjected to mechanical forces, such as soil compaction, the cell wall deforms. Information regarding this deformation is transmitted to the cell membrane through proteins that connect to the cell wall [31]. The cytoskeleton (microfilaments and microtubules) is connected to the cell membrane and can sense and transmit this mechanical signal. Dynamic changes in the cytoskeleton can act as sensors for mechanical signals, converting external mechanical stimuli into biochemical signals within the cell [54].

Some ion channels in the cell membrane, such as the calcium ion (Ca^2+^) channel, are mechanically sensitive [14]. When roots are mechanically stimulated, the tension of the cell membrane changes, which directly activates the mechanically sensitive ion channel. After the channel opens, Ca^2+^ outside the cell rapidly flows into the cell, resulting in an instantaneous increase in the intracellular Ca^2+^ concentration, thus converting the mechanical signal into a chemical signal [31].

### Generation and signal transmission of intracellular second messengers

Intracellular Ca^2+^ concentration increases early in the response of plant roots to mechanical stimuli. As a second messenger, Ca^2+^ can bind to calcium-binding proteins in the cell, such as calmodulin and calcium-dependent protein kinase [18]. After calmodulin binds to Ca^2+^, its conformation change allows it to interact with downstream target proteins to regulate the activities of various enzymes. For example, protein kinases and phosphatases can be activated, initiating a series of phosphorylation cascade reactions. Calcium-dependent protein kinase is activated after binding to Ca^2+^ and can directly phosphorylate downstream protein substrates, transmitting the signal and affecting gene expression and cellular physiological activity [56].

Mechanical stimuli can also trigger the production of ROS in root cells, which can act as signaling molecules to activate downstream signal transduction pathways. ROS can directly oxidize and modify proteins, thereby altering their activities and functions. ROS can also activate the mitogen-activated protein kinase (MAPK) cascade pathway [41]. This pathway consists of a series of protein kinases that transmit signals through step-by-step phosphorylation, ultimately activating transcription factors and regulating gene expression. NO is a signaling molecule that plays a role in the response of plant roots to mechanical stimuli [70]. Mechanical stimuli may activate an NO synthase-like pathway or a nitrate reductase pathway, leading to an increase in NO production in cells [30].

### Gene expression regulation and physiological responses

The signal transduction pathways explained above ultimately activate a series of transcription factors, which can bind to the promoter regions of specific genes to regulate their expression. For example, under mechanical stimulation, the expression of genes related to cell wall synthesis and remodeling, stress response, and growth regulation will change [91], leading to physiological responses. Roots will adjust the composition and structure of the cell wall to enhance its strength and toughness to adapt to mechanical pressure [49]. They regulate cell growth and division, changing the morphology and structure of the roots, such as by increasing root branching and altering the root growth direction. Roots also adjust their metabolic activities by increasing the synthesis of secondary metabolites to improve the antioxidant capacity and stress resistance of the roots (Table 2).

**Table 2:** Active substances in the pathways responding to mechanical stimulation and their biological effects

**Response pathway**	**Reference**	**Active substances**	**Specific effects**
**Ca** ^**2+**^ **dependent**	[34]	MSs, MSLs, OSCA, RMA, DEK1, CBPs	Repeated exposure to mechanical stimulation alters the morphology of plants, and mechanostimulated plants exhibit greater stress tolerance than naive plants.
	[28]	Piezo, JA, CAMTA1, CAMTA2, CAMTA3, TCH2, TCH4, Camta3	Piezo mechanosensitive ion channels have no major role in touch-induced gene expression and thigmomorphogenesis. The receptor-like kinase Feronia acts as a strong negative regulator of the JA-dependent branch of touch signaling.
	[64]	Ca^2+^, ROS, pH	Mechanical stimulation likely Ca^2+^-dependently activates RBOH C, causing ROS production in the cell wall. ROS production is coordinated with pH changes by the same transient Ca^2+^.
**Hormone regulation**	[72]	Omeprazole, ETH	Omeprazole as a proton pump inhibitor, enhances the mechanical stress-induced root growth reduction especially in lower pH media.
	[71]	ETH, SA, JA	Root-growth cessation in response to mechanical stress involves ethylene signaling. Salicylic acid, which inhibits ethylene biosynthesis, alleviates root growth reduction.
	[67]	IAA, wuschel related homeobox, lateral organ boundaries domain	Physical contact of leaf nodes with soil particles triggers transcriptional induction of potential auxin-responsive gene transcription factors, which are likely involved in the induction of AR formation.
	[42]	IAA, ETH, ABA, OsYUC8, OsAUX1	Ethylene uses auxin and ABA as downstream signals to modify rice root cell elongation and radial expansion, causing root tips to swell and reducing their ability to penetrate compacted soil.
	[90]	IAA, ACC, SRDX, VIP1	VIP1 can suppress the touch-induced root waving. VIP1 influences root cap structure and regulates local auxin responses in roots.
	[59]	ETR1, ERS1, ETR2, EIN4, ERS2, CTR1, EIN2, EIL1, AUX1, PINs, WDL4, ERF1, WDL5, PGX3, EIN3, WEI8, TAA1, ASA1, GY1, EIL2	Ethylene signaling regulates the growth and development of plants encountering physical barriers. Ethylene inhibits root elongation through induction of auxin biosynthesis.
**MAPK**	[55]	MAPK, MAPKK, MAPKKK	MAPK cascade is the common mechanism for translating extraneous environmental signals into molecular and cellular responses.
**RLKs, Ca** ^**2+**^ **dependent**	[62]	FERONIA (FER), Mechanosensitive channel of small conductance like, MID1-complementing activity	Cell wall conformational changes induced by mechanical cues are monitored by a plasma membrane sensor. Mechanotransduction may be mediated by phosphorelay pathways and calcium signaling.
	[63]	MSL family, AtHK1, ARR3, ARR4, ARR 8, ARR 9, RLKs, WAKs 51, WAKs 52, WAKs 53, THE1 59, THE1 60	Plant mechanosensors fall into two broad classes: those activated by tension in the membrane, and those monitoring wall status or sheer between wall and plasma membrane.
**RLKs, Hormones regulation**	[12]	JA, GA, GA2OX7, GID1, DELLA, rga-t2, gai-t6, rgl1-1, rgl2-1, rgl3-4, AOS, JAR1, COI1, MYC2, MYC3, MYC4, OPR3, JAZ	Loss-of-function in GA2ox7 and global della impair some mechanical stress-induced phenotypes of thigmomorphogenesis.
**MAPK, Ca** ^**2+**^ **dependent**	[92]	TREPH1	Protein phosphorylation and TREPH1 protein are critical for mechanotransduction pathway leading to plant thigmomorphogenesis.
**Ca** ^**2+**^ **dependent, Hormones regulation**	[100]	TCH2 (CML24-2 and CML24-4) and TCH3 (CML12-2)	CML24/12 promote root elongation and inhibit skewing to mechanical cues. CML24-2/4 regulate root growth, indirectly affecting skewing and elongation through gravity responses under mechanical stimulation.
	[65]	Ca2+, RLK, MCA, Piezo, CaM, CDPKs, CBLs, JA, MSL, SACC, CC	Ca^2+^ signaling regulates the expression of some mechanoresponsive genes and activates plasma membrane transport processes that could rapidly alter cell-wall extensibility and remodeling.
	[40]	MSLs, MCA1, AHK, ABA, ETH, IAA	Plant roots sense mechanical stimuli through altering the expression of membrane-located ion channel- or stimulus receptor-related genes. Several plant hormones restrict root deepening but promote root thickening.
	[38]	MSCs, RLKs, Ca2^+^, ROS, CaM, JA, GA, BRs, ETH, ABA, IAA	Calmodulin (CaM) decodes the Ca^2+^ signatures, which along with various hormones leads to transcription factor-mediated transcriptional changes that help shape the thigmomorphogenetic response.
	[95]	ETH, ABA, IAA, CRTISO, ZEP, NCED2, AAO2, OsYUC8, OsAUX1, OsEIL1, ROS, PIEZO, MCA1	Ethylene accumulation upregulates ABA biosynthesis and auxin biosynthesis and transport. ABA promotes radial root expansion. Increased auxin promotes root hair elongation, and ethylene enhances crown root number.
	[44]	ROS, RHD2, DPI, Ca^2+^, NADPH oxidase, HPCAs, H_2_O_2_	Inhibition of ROS signaling leads to early changes in root response to mechanical impedance, suggesting a requirement for ROS signaling in the activation of ethylene signaling. ROS production requires Ca^2+^ signaling.

**Figure 3:**
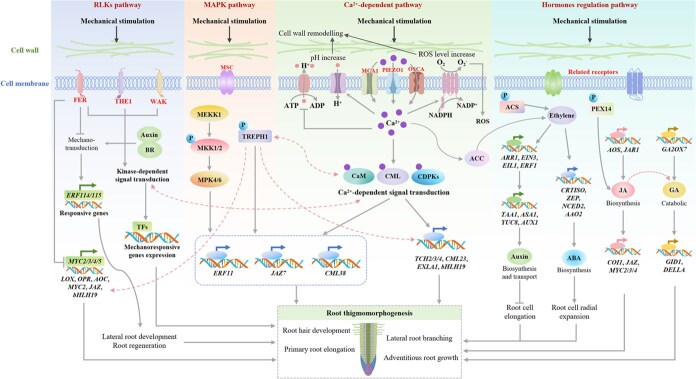
Molecular mechanisms underlying root responses to mechanical stimuli. This figure illustrates the molecular response of plant roots to mechanical stimuli. The response of plant roots to mechanical stimuli mainly occurs through four pathways: RLK (Receptor-like kinase), MAPK (mitogen-activated protein kinase), Ca^2+^-dependent, and hormone regulation pathways. Red dotted lines indicate potential effects.

In the RLKs pathway, FER (FERONIA receptor kinase), THE1 (THESEUS1), and WAK (Wall-associated kinase) are subjected to mechanical stimuli, triggering protein kinase-dependent signals (Figure 3). These signals transmit mechanical information to mechanically responsive genes or interact with Ca^2+^-dependent signal transduction [64]. Auxin and brassinosteroids (BR) promote the transmission of mechanical signals to the nucleus, and the transcription factors ERF114 and ERF115 activate the expression of responsive genes, whereas FER inhibits this signal transmission [62]. FER may also regulate the expression of downstream mechanical signals and related genes, such as lipoxygenases (*LOX*), 12-oxophytodienoate reductase (*OPR*), allene oxide cyclase (*AOC*), jasmonate ZIM-domain proteins (*JAZ*), and basic helix-loop-helix transcription factor 19 (*bHLH19*), by inhibiting the phosphorylation of transcription factors MYC2/3/4/5 [28]. In the MAPK pathway, mechanical stimuli increase the phosphorylation levels of touch-regulated phosphoprotein 1 (TREPH1) and mitogen-activated protein kinase kinase 1/2 (MKK1/2), transmitting mechanical signals downstream to regulate the expression of genes *ERF11, JAZ7,* and calmodulin-like 38 (*CML38*) [92].

In the Ca^2+^-dependent pathway, mechanical stimuli induce stress responses through several ion channels, including MCA1 (Mid1-complementing activity 1), PIEZO1 (piezo-type mechanosensitive ion channel component 1) [26, 27, 66], and OSCA (hyperosmolality-gated Ca^2+^-permeable channels), leading to Ca^2+^ influx in root cells [40, 63–65, 72]. The increase in cytoplasmic calcium ion concentration activates proton transporters, resulting in cytoplasmic acidification and cell-wall alkalization. Meanwhile, Ca^2+^ activates NADPH oxidase, generating ROS in the cell wall, and cell-wall ROS may flow back into the cytoplasm through the cell membrane [63, 64]. The increase in cell-wall pH and ROS leads to cell-wall remodeling. As a second messenger, Ca^2+^ binds to CaM (calmodulin), CML (calmodulin-like protein), and CDPKs (calcium-dependent protein kinases), transmitting mechanical signals downstream to regulate the expression of related genes [*TCH2/3/4* (touch inducible gene 2/3/4), *EXLA1* (expansin like gene 1), *CML23*, and *bHLH19*] ([64] a; [28, 65, 92]).

Mechanical stimuli induce ethylene accumulation, which upregulates the expression of TAA1 (tryptophan aminotransferase 1), ASA1 (anthranilate synthase alpha 1), YUC8 (indole-3-pyruvate monooxygenase 8), and AUX1 (auxin influx carrier auxin-resistant 1) through ARR1 (Arabidopsis response regulator 1), EIN3 (ethylene insensitive 3), EIL1 (EIN3-like 1), and ERF1, promoting the biosynthesis and transport of auxin and thus inhibiting root cell elongation [42, 59, 71, 95]. Ethylene accumulation also promotes the biosynthesis of ABA (abscisic acid) (including genes such as *CRTISO, ZEP, NCED2,* and *AAO2*), thereby promoting the radial expansion of root cells [42, 95]. Mechanical stimuli activate the expression of genes related to JA biosynthesis (*AOS* [hydroperoxide dehydratase] and *JAR1* [jasmonate-resistant 1]) and JA signal transduction (*COI1* [coronatine-insensitive 1], *JAZ*, and *MYC2/3/4*), increasing the intracellular JA level [12, 94]. Mechanical stimuli may also promote gibberellin decomposition by upregulating the expression of *GA2OX7* (GA2-oxidase gene 7), while regulating the expression of GID1 (GIBBERELLIN-INSENSITIVE DWARF 1) and DELLA protein, ultimately affecting root contact morphogenesis [12].

### Interaction and integration of intracellular signaling networks

During the signal transduction process of plant roots in response to mechanical stimuli, complex interactions occur between different signaling molecules. For example, Ca^2+^, ROS, NO, and plant hormones do not function independently but are intertwined to form a complex signaling network. Ca^2+^ signal can regulate ROS production and scavenging (Monshausen and Gilroy, 2009a). Ca^2+^ can activate enzymes related to ROS metabolism, such as NADPH oxidase, to promote ROS production. At the same time, calmodulin can regulate the activity of antioxidant enzymes, thereby affecting ROS scavenging. There is a synergistic or antagonistic relationship between NO and ROS. In some cases, NO can react with superoxide anions to form peroxynitrite anions, and this reaction can affect the intracellular redox state and signal transduction. In other cases, NO can reduce ROS damage to cells by regulating the activity of antioxidant enzymes [79]. Plant hormones also interact with other signaling molecules. The transport and signal transduction of auxin are regulated by ROS and NO. ROS can affect the localization and activity of auxin transport carriers, thereby altering the distribution of auxin. NO can interact with key proteins in the auxin signaling pathway to regulate the response to auxin [74].

Crosstalk between different signal transduction pathways helps to achieve an accurate response to mechanical stimuli. For example, the MAPK cascade pathway can be activated not only by ROS but also by interacting with calcium ion signals and plant hormone signaling pathways [81]. Under mechanical stimulation, the MAPK cascade pathway regulates the activity of transcription factors through phosphorylation, and these transcription factors may be simultaneously regulated by calcium–calmodulin complexes and plant hormone signals [99]. Crosstalk also exists between the ethylene and auxin signaling pathways [44]. This crosstalk enables plants to coordinate the growth and development of roots according to changes in the mechanical environment.

## Technological Progress, application prospects, and future research on response of plant roots to mechanical environmental stimuli

### Challenges and technological Progress

**Figure 4:**
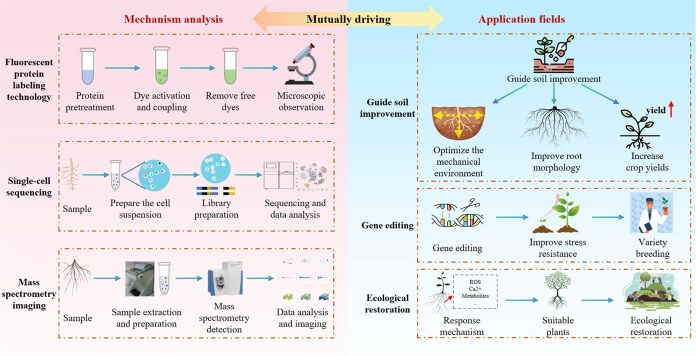
Advanced signal detection technologies and application prospects.

Advanced techniques have been applied to the research of mechanical signal transduction in plant roots, greatly promoting the development of this field. By using fluorescent proteins to label intracellular signaling molecules, such as Ca^2+^ and ROS, real-time and dynamic changes in the concentration and distribution of these signaling molecules within cells can be observed [20] (Figure 4). Through mass spectrometry, changes in the content of signaling molecules, such as auxin, ethylene, and NO, in plant roots under mechanical stimulation [44], as well as the synthesis and accumulation of secondary metabolites, can be accurately determined, providing information for studying the downstream effects of signal transduction [4].

Multidisciplinary research has led to breakthroughs in our understanding of plant root mechanical responses; however, several key issues remain. The core processes of mechanical signal perception and transduction remain unclear, mechanisms underlying mechanical responses with other stress signaling pathways require further analysis, and current research methods struggle to capture real-time, in-situ dynamic interactions between roots and mechanical forces. Future research should aim to use single-cell RNA sequencing [22] to analyze the specific gene expression of different root cell types (e.g., root cap and meristematic zone) under mechanical stimulation, study the coordination and antagonism of hormone signals, and identify key proteins and ion channels. Spatial transcriptomics can be applied to locate the responding genes and tissue regions, and spatial metabolomics can be used to observe the in situ changes in hormones and cell wall components, revealing the dynamics of cell wall remodeling. Fluorescent protein labeling can be adopted to monitor real-time changes in proteins, capture signaling events, and verify the interaction between mechanosensitive receptors and downstream signals. Non-invasive micro-test technology can be utilized to detect real-time changes in the ion/molecule fluxes in different root micro-regions and analyze the redirection of auxin flow and other response mechanisms.

### Application prospects

In-depth research on mechanical signal transduction in plant roots can provide new ideas and methods for agricultural production. Understanding the signal transduction mechanisms that underlie root response to the mechanical environment can guide soil improvement measures (Figure 4). For example, by adjusting the physical properties of soil, such as compaction and particle composition, the mechanical environment for root growth can be optimized to promote root growth and development [61]. Plant growth regulators can be used to regulate key signaling molecules in the root signal transduction pathway, such as auxin and ethylene [44], to control root growth and morphology and improve the stress resistance and yield of crops.

Through biotechnological methods, such as gene editing, genes related to root signal transduction can be modified and regulated to breed crop varieties with better mechanical adaptability [10]. For example, enhancing the tolerance of roots to soil compaction will enable crops to grow under relatively poor soil conditions, thereby reducing the yield losses caused by unfavorable soil mechanical properties [39, 95].

Research on mechanical signal transduction in plant roots can also have implications for ecological restoration (Figure 4). By improving the mechanical properties of the soil and providing appropriate mechanical stimuli, the growth and development of plant roots can be promoted and the survival rate and restoration effect of vegetation can be improved [32].

As shown as Figure 4, the continuous development of advanced technologies, such as fluorescent protein labeling, single-cell sequencing, and mass spectrometry imaging, provides technical support for the precise detection of signaling molecules, such as calcium ions, reactive oxygen species, nitric oxide, and plant hormones, as well as for in-depth exploration of the physiological and biochemical mechanisms of plant roots in response to mechanical stimuli. Adjusting the physical and chemical properties of soil according to the response mechanism of roots to mechanical stimuli can guide soil improvements. Screening key genes related to the mechanical response of roots can help cultivate varieties with strong stress resistance. Selecting appropriate plant species to promote better root growth and function in damaged soil can accelerate ecosystem restoration.

### Future research

#### Multi-signal integration mechanism in root Thigmomorphogenesis response

Various signaling molecules, including Ca^2+^, auxin, ROS, and electrical signals, are involved in the thigmomorphogenesis response of roots. However, how these signals act synergistically to produce a unified and precise growth response remains unclear. This is a key issue that urgently needs to be addressed in future studies, which should focus on the identification of key integration nodes and spatio-temporal dynamic analysis of signal decoding.

Firstly, regarding the identification of key integration nodes, attention should be directed toward protein kinases (such as CDPKs and MAPKs) and phosphatases that are co-regulated by Ca^2+^ signals (e.g., calmodulin-like proteins CMLs), ROS, and pH changes. These enzymes serve as central platforms for signal cross-talk. Through phosphorylation/dephosphorylation modifications, they integrate multiple upstream signals and regulate the activity and polar localization of downstream auxin transport carriers (such as PIN proteins). In-depth research on the association between the initially identified mechanosensitive receptors (such as MCA and FERONIA receptor kinases) and the cytoskeleton is also required. Future studies should aim to elucidate how the dynamic reorganization of microfilaments influences the spatio-temporal oscillations of Ca^2+^ signals under mechanical stimulation and how the two jointly guide the direction of auxin.

Secondly, in exploring the spatio-temporal dynamics of signal decoding, advanced techniques such as in-vivo imaging and FRET biosensors can be employed to visualize real-time changes in multiple signals (including Ca^2+^, auxin, pH, and ROS), as roots interact with obstacles. Such approaches would help to reveal the sequence of signal transmission, feedback loops, and specific patterns in different cell types (such as the root cap, elongation zone, and meristematic zone) and provide a high-resolution signal roadmap.

#### Targeted cultivation of crops adaptable to mechanical stimuli using gene editing technology

Once the above signal pathways are clarified, gene-editing technologies, such as CRISPR/Cas, can be applied to breed crops that can better adapt to compacted and gravelly soil environments (Figure 4). Potential targets include the optimization of mechanical signal perception and transduction components by editing genes encoding mechanosensitive receptors (e.g., *MCA*) to generate allelic variants with enhanced sensitivity to soil mechanical resistance. Such modifications would enable roots to detect obstacles more rapidly and initiate timely avoidance responses. Editing genes that regulate Ca^2+^ signal oscillations (e.g., Ca^2+^ channels or pumps) could fine-tune their amplitude and frequency, potentially optimizing signal transmission efficiency and accuracy. The auxin response and transport network could be reshaped by editing the promoter or coding region of PIN-FORMED (PIN) auxin efflux carrier genes to change their expression or polar distribution on specific root-tip cell surfaces. This could program roots to develop architectures that expand horizontally or penetrate deeply. Editing negative regulatory factors in the auxin signaling pathway (e.g., Aux/IAA proteins) can make them more degradable under mechanical stimulation, enhancing the auxin response rate and making root bending responses more agile. As root adaptability is a complex trait controlled by multiple genes, future breeding strategies should not be limited to single-gene editing. By simultaneously editing multiple genes within a signal module (e.g., receptor gene + Ca^2+^ regulator + *PIN* gene), the synergistic enhancement of root functions can cultivate a new generation of intelligent crops with higher resilience and yield in poor soil. Studies on the specificity of root responses in different plant species must expand the sample scope to include more representative species and use bioinformatics and systems biology methods to explore the underlying genetics and evolutionary mechanisms. Such research will achieve a substantial breakthrough at the conceptual level and provide new theoretical frameworks and research perspectives for this field.
